# Total Sleep Time in the Taiwan Obstructive Lung Disease Cohort

**DOI:** 10.3390/ijerph18137080

**Published:** 2021-07-02

**Authors:** Li-Pang Chuang, Meng-Jer Hsieh, Ning-Hung Chen, Han-Chung Hu, Cheng-Ta Yang, Ying-Huang Tsai, Shih-Wei Lin

**Affiliations:** 1 Sleep Center and Department of Pulmonary and Critical Care Medicine, Chang Gung Memorial Hospital, Linkou 33305, Taiwan; r5243@cgmh.org.tw (L.-P.C.); mengjer@cgmh.org.tw (M.-J.H.); ninghung@yahoo.com.tw (N.-H.C.); h3226@cgmh.org.tw (H.-C.H.); yang1946@cloud.cgmh.org.tw (C.-T.Y.); 2 School of Medicine, Chang Gung University, Taoyuan 33302, Taiwan; 3 Department of Respiratory Therapy, Chang Gung University, Taoyuan 33302, Taiwan; 4 Department of Pulmonary and Critical Care Medicine, Xiamen Chang Gung Hospital, Xiamen 361028, China; chestmed@cloud.cgmh.org.tw

**Keywords:** comorbidity, COPD, PSQI, pulmonary function, total sleep time

## Abstract

Patients with chronic obstructive pulmonary disease (COPD) have been reported to have poor sleep quality. However, total sleep time has not been evaluated in detail among patients with COPD. This retrospective, observational, multicenter research study was performed across six participating hospitals in Taiwan, with a total of 421 adult patients enrolled. Pulmonary function, the Modified British Medical Research Council Dyspnea Scale, the COPD Assessment Test and basic clinical data were assessed. The Pittsburgh Sleep Quality Index was also administered to patients, and the total sleep time was extracted for further analysis. The patients whose total sleep time was between 6 and 7 h had better pulmonary function, and the patients who slept less than 5 h had worse comorbidities. There was a significant higher total sleep time in Global Initiatives for Chronic Obstructive Lung Disease (GOLD) group B compared to GOLD group A. COPD patients who sleep between 5 and 6 h used fewer oral steroids and were less likely to use triple therapy (long-acting beta-agonist, long-acting muscarinic antagonist, inhaled cortical steroid). COPD patients sleeping from 5 to 7 h had better clinical features than those sleeping less than 5 h in terms of pulmonary function, comorbidities and medication usage.

## 1. Introduction

Sleep quality is impaired in patients with chronic obstructive pulmonary disease (COPD), as these patients have higher levels of sleep fragmentation and decreased slow-wave and rapid-eye movement (REM) stages [1,2]. Night-time symptoms have also been reported incrementally with the severity of airflow limitation in COPD patients [3]. Although sleep disturbance is a common symptom in patients with COPD compared to the general population [4,5,6], people do not always pay much attention to the night-time symptoms of COPD, as indicated by the gap in the self-report rate of night-time symptoms between physicians and patients [7].

Oxygen desaturation often occurs during sleep in COPD patients with less REM sleep and arousal during that period [2]. Some studies have shown that COPD patients suffer from hypoxemia during sleep at a higher level than during exercise [8]. However, in patients with COPD, the severity of nocturnal oxygen desaturation and airflow limitation, presented with forced expiratory volume in the first second (FEV_1_) were weakly correlated with sleep quality [9,10].

Patients with COPD not only report poor sleep quality but also report short total sleep time [11]. A short total sleep time may indicate that the COPD patient slept for an insufficient period of time due to physiological reasons, certain medical conditions or sleep disorders [12]. One study showed that nocturnal non-invasive positive pressure ventilation (NIPPV) can increase the total sleep time in hypercapnic COPD patients [13]. Some studies also reported the influence of medication on total sleep time in COPD patients [14,15]. According to previous studies, people with a short sleep time may suffer from a higher mortality and morbidity of cardiovascular events [16]. In a meta-analytic study, a U-shaped association between sleep duration and mortality was reported, in which short sleepers have a 12% greater risk of mortality than those who sleep from 7 to 8 h per night [17].

Our previously published data in a Taiwanese Obstructive Pulmonary Disease (TOLD) cohort revealed that 53% of COPD patients have poor sleep quality, as indicated by Pittsburg Sleep Quality Index (PSQI) scores greater than 5 [18]. Total sleep time has been reported to be significantly correlated with subjective sleep quality, and previous reports show that a short or long sleeper may suffer from a higher mortality and morbidity [16,19]. Thus, in this post hoc analysis, we aim to describe the total sleep time and pulmonary function in COPD patients.

## 2. Materials and Methods

### 2.1. Clinical Patients

This retrospective, observational, multicenter research study was performed across six participating hospitals in Taiwan, with a total of 421 patients [20]. Adult COPD patients over 40 years old who were followed in an outpatient pulmonary clinic were enrolled between December 2011 and November 2013. This study was conducted in accordance with the amended Declaration of Helsinki and approved by the Institutional Review Boards of all the hospitals involved, and written informed consent was obtained from each participant before enrolling. The COPD diagnosis, based on the Global Initiatives for Chronic Obstructive Lung Disease (GOLD) guidelines, was used [21]. All participants had pulmonary function tests within one year and completed the PSQI at least once [22]. In the GOLD guidelines that were updated in 2011, COPD patients are classified into four groups by their symptoms and lung function [21]. COPD symptoms were evaluated by the Modified British Medical Research Council (mMRC) Dyspnea Scale or the COPD Assessment Test (CAT) as follows: patients with fewer symptoms (group A or C): mMRC 0–1 or CAT < 10; patients with more symptoms (group B or D): mMRC ≥ 2 or CAT ≥ 10; low-risk patients (group A or B): FEV_1_% predicted ≥50% or COPD exacerbation history 0–1 time in the past year; high-risk patients (group C or D): FEV_1_% predicted <50% or COPD exacerbation history ≥2 times in the past year. The patients with COPD were categorized into groups A, B, C, and D.

### 2.2. General Data Collection

The general data were retrospectively collected from medical records, including age, gender, body weight, body height, body mass index (BMI), smoking status, COPD medications prescribed at outpatient clinics, clinical symptoms, pulmonary function tests, CAT scores [23], mMRC Dyspnea Scale scores [24], and medical history of comorbidities. The COPD maintenance inhaler was defined as the continuous prescription of an inhaler for at least 3 months.

### 2.3. Measurement of Total Sleep Time

The PSQI is a self-reported screening tool that measures quality of sleep. It assesses seven components of sleep, including sleep quality, sleep latency, sleep duration, sleep efficiency, sleep disturbances, the use of sleeping medication, and daytime dysfunction. Each component is scored from 0 to 3, and seven component scores are then summed to obtain a global score [22]. The traditional Chinese version of the PSQI was used to assess sleep quality in this study, with the permission of the original author [25]. The results of the PSQI were retrospectively collected and analyzed. The self-reported total sleep time was extracted from the PSQI questionnaire, and patients were divided into four groups according to the scale of the PSQI score given, including patients who sleep more than 7 h per night, between 6 and 7 h per night, between 5 and 6 h per night and less than 5 h per night.

### 2.4. Statistical Analysis

Patient demographics, clinical characteristics (including total sleep time), and medications were summarized using descriptive statistics. A *t*-test was used to compare mean values between two groups, and one-way analysis of variance (ANOVA) was used to compare differences among more than two groups. A directed acyclic graph (DAG) was drawn to identify the minimum set of confounders using the R package ‘DAGitty’ [26]. Linear regression was used to look at the association between total sleep time and lung function based on the minimum set of confounders. All statistical tests were performed with SPSS software (SPSS Institute, Chicago, IL, USA). A *p* value of 0.05 or less is considered to indicate statistical significance, and all data are expressed as the mean ± standard deviation (SD).

## 3. Results

The PSQI data revealed that over 53% of our COPD patients have poor sleep quality as indicated by PSQI global scores greater than 5. In regard to the seven components of PSQI, “sleep disturbance” achieved the highest score (mean ± SD = 1.5 ± 0.6) compared with the other components; and the most common sleep disturbances were “getting up to use the bathroom (70%)”, “wake up at night or early morning (40%)” and “cough and snore loudly at night (16% and 12%, respectively)” (data retrieval from [20] under the permission of the original author) [18]. Table 1 shows the general basic data from patients according to four different total sleep times: more than 7 h (mean ± SD = 8.4 ± 0.6), between 6 and 7 h (mean ± SD = 6.8 ± 0.1), between 5 and 6 h (mean ± SD = 5.6 ± 0.2), and less than 5 h (mean ± SD = 3.8 ± 0.6). There was no statistical significance between the four groups in age, gender, BMI or smoking history (Table 1).

**Figure 1:**
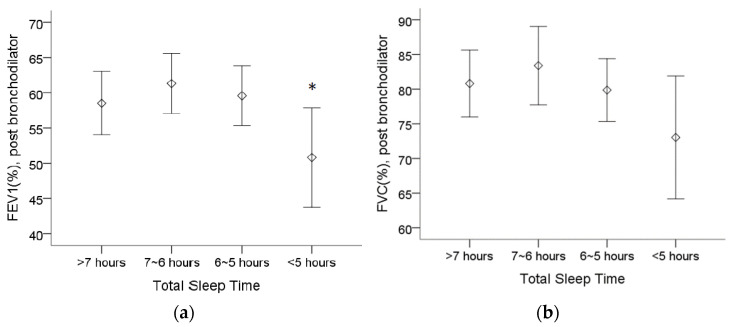
The assessment of pulmonary function in 4 different total sleep time groups from spirometry. (**a**) The post bronchodilator predicted forced expiratory volume in 1 s (FEV_1_(%)) was lower in patients who sleep less than 5 h than in patients who sleep between 6 and 7 h. (**b**) The trend of lower forced vital capacity (FVC (%)) appeared in patients who sleep less than 5 h, although no statistical significance was found. Note: 95% confidence interval, *: *p* < 0.05 vs. 6~7 h. Abbreviations: FEV_1_ = forced expiratory volume in one second, FVC = forced vital capacity.

Table 2 shows that COPD patients who sleep less than 5 h have a worse FEV_1_ (%), whereas patients who sleep between 6 and 7 h have the highest FEV_1_ and force vital capacity (FVC). The post bronchodilator predicted FEV_1_ (%) was lower in patients who sleep less than 5 h than in patients who sleep between 6 and 7 h (Figure 1a). Although there was no statistically significant difference regarding the post bronchodilator predicted FVC (%), we observed a trend of a lower FVC (%) in patients who sleep less than 5 h (Figure 1b).

**Figure 2:**
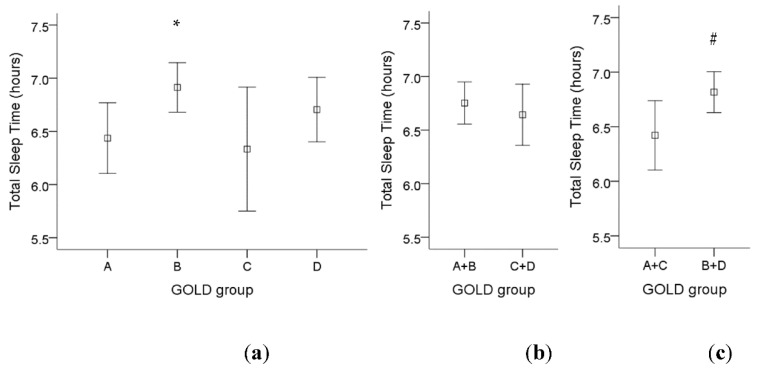
The influence of different GOLD groups on total sleep time in COPD patients. (**a**) There was a significant increase in total sleep time in GOLD group B compared to GOLD group A. (**b**) There was no significant difference in total sleep time among low-risk patients (group A and B) compared to high-risk patients (group C and D). (**c**) There was a significant higher total sleep time in COPD patients with more symptoms (group B and D) compared to fewer symptoms (group A and C). Note: 95% confidence interval, *: *p* < 0.05 vs. Group A; #: *p* < 0.05 vs. Group A + C. Abbreviations: GOLD = Global Initiative for Chronic Obstructive Lung Disease.

For GOLD criteria, there was a significant higher total sleep time in GOLD group B compared to GOLD group A (Figure 2a). If we divided those patients into fewer symptoms (group A and C) or more symptoms (group B and D), there was a significant higher total sleep time in COPD patients with more symptoms (Figure 2c). However, there was no significant difference in total sleep time among low-risk patients (group A and B) compared to high-risk patients (group C and D) (Figure 2b).

According to the reported comorbidities of those COPD patients, there were no statistically significant differences in cardiovascular disease, such as hypertension, ischemic heart disease, heart failure and arrhythmia, between the different sleep time groups (Table 3). However, regarding metabolic diseases, such as diabetes mellitus, dyslipidemia and osteoporosis, patients who sleep for less than 5 h have a higher percentage of those diseases compared to other groups.

**Figure 3:**
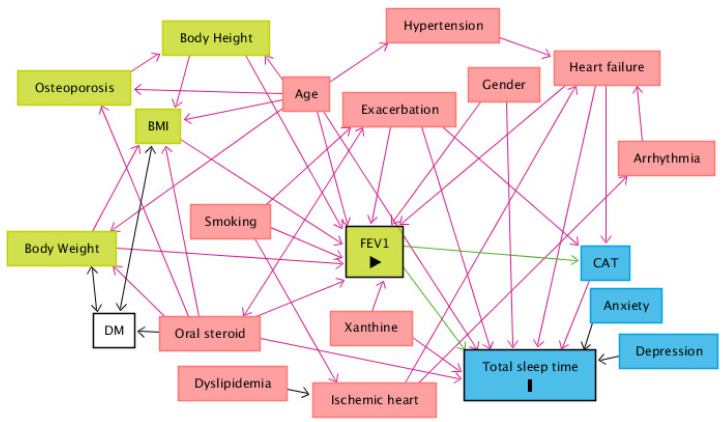
A directed acyclic graph (DAG) was drawn to identify the minimum set of confounders. The minimal sufficient adjustment sets for estimating the direct effect of FEV_1_ on total sleep time are age, CAT, exacerbation, gender, heart failure, oral steroids and xanthine. Abbreviations: BMI = body mass index; CAT = COPD Assessment Test; DM = diabetes mellitus; FEV_1_ = forced expiratory volume in one second. Legends: green square = ancestor of exposure; blue square = ancestor of outcome; pink square = ancestor of exposure and outcome; transparent square = other variables; green arrow = causal path; pink arrow = biasing path.

When comparing the use of medication among sleep time groups, patients who sleep between 5 and 6 h tend to use less maintenance inhaled triple therapy (long-acting beta-agonist (LABA) combined with long-acting muscarinic antagonist (LAMA) and inhaled cortical steroid (ICS)) than patients who sleep less than 5 h and patients who sleep more than 7 h. Patients who sleep between 5 and 6 h also use fewer oral steroids than the other groups (Table 4). Finally, after drawing a DAG to identify the minimum set of confounders, the minimal sufficient adjustment sets for estimating the direct effect of FEV_1_ on total sleep time are age, CAT, exacerbation, gender, heart failure, oral steroids and xanthine (Figure 3). The corresponding regression analysis, based on the minimum set of confounders, revealed no significant association between total sleep time and FEV_1_ (Coefficient = 0.028, *p* = 0.896) (Table 5).

## 4. Discussion

This study is the first to describe the impact of sleep duration on COPD. The PSQI was administered to COPD patients and revealed that patients whose total sleep time was between 6 and 7 h had higher pulmonary function and fewer comorbidities than patients in other sleep time groups.

The total sleep time is the amount of actual sleep time in a sleep episode [12]. Although total sleep time is only one parameter of measurable sleep characteristics, it has been reported to be significantly correlated with subjective sleep quality [19]. Several previous reports, including one by Kripke et al., show that a short or long sleeper in the general population may suffer from a higher mortality and morbidity of cardiovascular events [16]. A U-shaped association between sleep duration and mortality was reported in a prior meta-analytic study, in which short sleepers (<7 h per night) have a 12% greater risk of mortality and long sleepers (>8 h per night) have a 30% greater risk of dying than those who sleep 7 to 8 h per night [17]. A low total sleep time may indicate that the patient slept for an insufficient period of time due to physiological reasons, or certain medical or sleep disorders, and a long total sleep time may suggest prior sleep deprivation, medical conditions, or effects of medications [12]. As we know, sleep apnea is common in COPD patients and results in sleep fragmentation and nonrestorative sleep [27]. Although high levels of sleep fragmentation may result in complaints of nonrestorative sleep even when an apparently normal total sleep time is present, sleep fragmentation does not explain misperception of total sleep time [28].

Sleep disturbance is a common symptom in patients with COPD [4,5,6]. Patients with COPD exhibited relatively normal daytime sleepiness, despite a short total sleep time and numerous arousals from sleep [11]. In patients with moderate to severe COPD, nocturnal desaturation was not correlated with sleep quality or quality of life [9]. However, in another study, disturbed sleep was correlated with cough and dyspnea symptoms but not with FEV_1_ in patients with COPD [10]. On the other hand, some of the literature has shown that poor sleep quality, especially insomnia, can lead to poor COPD outcome [29]. Previous research investigating the bidirectional longitudinal associations between sleep disturbance and the severity of COPD concluded that the disease severity predicts future sleep disturbance in COPD patients [30]. Thus, it is worth studying the characteristics shared between total sleep time and clinical features of COPD. It could be bidirectionally that poor sleep makes COPD worse, or worse COPD causes worse sleep. Although, after linear regression in our study, the FEV_1_ had no statistical significance with total sleep time after adjusting parameters with age, gender, BMI, smoking status or comorbidities, it demonstrated the complexity of factors which can influence the pulmonary function of COPD patients. Since our study is a cross-sectional study, we could not claim the causal relationship between total sleep time and clinical features of COPD.

Sleep disturbance is associated with inflammatory disease risk and all-cause mortality [17]. The associations between sleep duration and inflammation parallel the findings linking sleep and mortality [31]. Shorter sleep duration was associated with higher levels of CRP, and extremely long sleep duration was associated with higher levels of CRP and IL-6 [32]. However, not all kinds of inflammatory diseases are associated with sleep duration. In one report, the total sleep time, determined by wrist actigraphy, was not associated with incident hypertension [33]. Our study revealed that COPD patients who sleep less than 5 h have a higher percentage of metabolic diseases, such as diabetes, dyslipidemia and osteoporosis, but not hypertension or cardiac disease.

Commonly used inhaled bronchodilators, such as inhaled anticholinergic agents, beta-agonists and steroids, have been reported to influence the total sleep time in patients with COPD [15,34,35,36,37,38]. One early study revealed that ipratropium bromide therapy can improve sleep SaO_2_ but has no effect on total sleep time [14]. Another study showed that long-acting inhaled anticholinergic therapy improved sleeping oxygen saturation but had no influence on sleep architecture, including total sleep time [15]. However, in a recent study, inhaled anticholinergic agents were shown to improve all sleep architecture parameters, such as total sleep time, sleep efficiency, REM stage and slow-wave sleep stage [3,39]. Our study showed that COPD patients who sleep between 5 and 6 h seem to take less maintenance inhaled triple therapy (LABA + LAMA + ICS) and fewer oral steroids. However, a prior meta-analytic study found that, generally, sleeping from 7 to 8 h per night is ideal, compared to less than 7 h or more than 8 h [17]. Our study shows that the COPD patients might require less total sleep time. This phenomenon may be due to the better health status and lung function, due to the lower amounts of medication used by patients who slept between 5 and 6 h, compared to those who slept more than 7 h. Another possible reason for this is that our COPD patients are relatively older than the average age of the general population.

There are still some limitations in our study. The total sleep time used in our study is based on a standard questionnaire but not objective polysomnography (PSG) data. This may lead to some overestimation or underestimation of total sleep time from the report of the patient. However, as we know, the PSQI is a well-validated questionnaire that provides a relatively accurate measurement of total sleep time, and we suggest that it can still represent the sleep duration in our COPD patient [40]. Another issue is that we did not record the usage time of medicine, such as LABA inhaled in the morning or LAMA inhaled at night. Although most of the long-acting inhaled agents were used in the morning, we still could not neglect the circadian influence of the actual usage time. In addition, there are some sleep disorders, such as sleep apnea, that influence the total sleep time, which were not excluded in this study. Finally, since our study is a cross-sectional study, we could not state the causal relationship between total sleep time and clinical features of COPD. Further longitudinal research with PSG needs to be carried out in the future to rule out the influence of other sleep disorders and determine the relationship between total sleep time and COPD.

## 5. Conclusions

In this Taiwanese Obstructive Lung Disease cohort, COPD patients who sleep for less than 5 h have worse FEV_1_% and comorbidities, and use more medications. COPD patients who sleep between 6 and 7 h have better pulmonary function (FEV_1_ and FVC). COPD patients who sleep between 5 and 6 h use less maintenance inhaled triple therapy and fewer oral steroids. This pilot descriptive analysis implies that we need to pay more attention to the total sleep time in the clinical care of COPD patients. It seems that sleeping 5–7 h may be ideal for COPD patients, but further research is required to tease out the bi-directional associations.

## Figures and Tables

**Table 1:** Basic data from four groups according to total sleep time.

	Total Sleep Time				*p*-Value
	>7 h	6~7 h	5~6 h	<5 h	
No.	129	127	129	26	
Gender (male/female)	128/1	126/1	126/3	25/1	0.474
Age (y)	73.9 ± 9.5	71.8 ± 11.3	72.5 ± 9.3	70.7 ± 8.3	0.078
BMI (kg/m^2^)	23.6 ± 4.1	23.3 ± 3.7	23.6 ± 3.8	24.5 ± 4.3	0.573
Smoking history					0.261
Never smoker, n (%)	9 (7.1)	6 (4.9)	5 (4.1)	4 (15.4)	
Ex-smoker, n (%)	81 (63.8)	74 (60.2)	73 (59.8)	11 (42.3)	
Current smoker, n (%)	37 (29.1)	43 (34.9)	44 (36.1)	11 (42.3)	

Note: Data was present with mean ± SD, or number (%). Abbreviations: BMI = body mass index.

**Table 2:** Pulmonary function from four groups according to total sleep time.

Pulmonary Function	Total Sleep Time				*p*-Value
	>7 h	6~7 h	5~6 h	<5 h	
Pulmonary function					
FEV_1_, predicted (%)	58.5 ± 23.3	61.3 ± 22.7	59.6 ± 22.8	50.8 ± 16.7 *^b^*	0.222
FVC, predicted (%)	80.8 ± 24.9	83.4 ± 30.2	79.9 ± 24.3	73.0 ± 21.0	0.346
FEV_1_ (L)	1.29 ± 0.45	1.44 ± 0.51 *^a^*	1.39 ± 0.56	1.23 ± 0.59	0.081
FVC (L)	2.32 ± 0.58	2.50 ± 0.65 *^a^*	2.43 ± 0.77	2.23 ± 0.80	0.135
Exacerbations in previous year (times)	0.49 ± 0.09	0.64 ± 0.12	0.54 ± 0.10	0.69 ± 0.21	0.695
mMRC, mean ± SD	1.73 ± 0.87	1.72 ± 0.83	1.79 ± 1.06	1.98 ± 0.84 *^ab^*	0.082
CAT, mean ± SD	10.28 ± 6.60	9.90 ± 6.71	10.34 ± 7.46	12.04 ± 7.50	0.565

Note: Data was present with mean ± SD, *^a^*: *p* < 0.05 vs. >7 h, *^b^*: *p* < 0.05 vs. 6~7 h. Abbreviations: CAT = COPD Assessment Test, FEV_1_ = forced expiratory volume in one second, FVC = forced vital capacity, mMRC = Modified British Medical Research Council.

**Table 3:** Comorbidities from four groups according to total sleep time.

Comorbidities	Total Sleep Time				*p*-Value
	>7 h	6~7 h	5~6 h	<5 h	
Hypertension, n (%)	40 (28.0)	35 (26.5)	38 (26.0)	11 (36.7)	0.684
Ischemic heart disease, n (%)	13 (9.1)	10 (7.6)	8 (5.5)	2 (6.7)	0.702
Heart failure, n (%)	5 (3.5)	3 (2.3)	4 (2.7)	3 (10)	0.190
Arrhythmia, n (%)	3 (2.1)	6 (4.5)	7 (4.8)	1 (3.3)	0.626
Diabetes, n (%)	18 (12.6)	11 (8.3)	16 (11.0)	8 (26.7) *^bc^*	0.044
Dyslipidemia, n (%)	8 (5.6)	2 (1.5)	6 (4.1)	3 (10.0) *^b^*	0.136
Osteoporosis, n (%)	5 (3.5)	2 (1.5)	1 (0.7)	3 (10.0) *^bc^*	0.016
Depression, n (%)	2 (1.4)	1 (0.8)	6 (4.1)	1 (3.3)	0.230
Anxiety, n (%)	1 (0.7)	2 (1.5)	2 (1.4)	0 (0)	0.840

**Note:** Data was presented with number (%), *^b^*: *p* < 0.05 vs. 6~7 h, *^c^*: *p* < 0.05 vs. 5~6 h.

**Table 4:** Medication from four groups according to total sleep time.

Medication	Total Sleep Time				*p*-Value
	>7 h	6~7 h	5~6 h	<5 h	
Maintenance inhaler					
LABA, n (%)	11 (7.7)	10 (7.6)	9 (6.2)	1 (3.3)	0.814
LAMA, n (%)	28 (19.6)	37 (28.0)	34 (23.3)	6 (25.0)	0.402
LABA/LAMA, n (%)	9 (6.3)	9 (6.8)	11 (7.5)	1 (3.3)	0.862
LABA/ICS, n (%)	30 (21.0)	18 (13.6)	35 (24.0) ^b^	5 (16.7)	0.165
LAMA/ICS, n (%)	1 (0.7)	1 (0.8)	1 (0.7)	0 (0)	0.947
LABA/LAMA/ICS, n (%)	46 (32.2)	35 (26.5)	28 (19.2) ^ac^	11 (36.7)	0.047
No maintenance inhaler, n (%)	20 (14.0)	22 (16.7)	28 (19.2)	6 (20.0)	0.658
Oral steroids, n (%)	7 (4.9)	8 (6.0)	1 (0.7) ^abc^	2 (6.7)	0.090
Xanthine, n (%)	65 (59.6)	59 (62.1)	72 (69.2)	17 (73.9)	0.355
Carbocysteine, n (%)	12 (11.4)	12 (13.2)	14 (14.6)	3 (13.6)	0.931

Note: Data were present with number (%), *^a^*: *p* < 0.05 vs. >7 h, *^b^*: *p* < 0.05 vs. 6~7 h, *^c^*: *p* < 0.05 vs. <5 h. Abbreviations: ICS = inhaled cortical steroid, LABA = long-acting beta-agonist, LAMA = long-acting muscarinic antagonist.

**Table 5:** Regression of total sleep time on FEV_1_ with minimal adjusted covariates.

Covariates		Direct Effect of FEV_1_ on Total Sleep Time		
		Estimated Coefficient	Standard Deviation	*p*-Value
	Intercept	7.632	1.318	<0.001
	FEV_1_	0.028	0.214	0.896
Minimal adjusted covariates	Age	0.008	0.011	0.479
	CAT	−0.002	0.016	0.913
	Exacerbation	−0.068	0.098	0.487
	Gender	−1.228	0.849	0.150
	Heart Failure	0.553	0.521	0.290
	Oral Steroid	−2.614	1.041	0.013
	Xanthine	−0.332	0.235	0.159

Abbreviations: CAT = COPD Assessment Test; FEV_1_ = forced expiratory volume in one second.
